# Sex Differences in Association of Elevated Blood Pressure with Variables Characterizing Cardiometabolic Risk in Young Subjects with or Without Metabolic Abnormalities

**DOI:** 10.3390/ijerph17103612

**Published:** 2020-05-21

**Authors:** Katarína Šebeková, Radana Gurecká, Melinda Csongová, Ivana Koborová, Jozef Šebek

**Affiliations:** 1 Institute of Molecular BioMedicine, Faculty of Medicine, Comenius University, 811 08 Bratislava, Slovakia; radana.kollarova@gmail.com (R.G.); melinda.csongova@gmail.com (M.C.); koborova@gmail.com (I.K.); 2 Institute of Medical Physics, Biophysics, Informatics and Telemedicine, Faculty of Medicine, Comenius University, 811 08 Bratislava, Slovakia; 3 Institute of Materials & Machine Mechanics, Slovak Academy of Sciences, 845 13 Bratislava, Slovakia; sebek.jozef@savba.sk

**Keywords:** adolescents, cardiometabolic risk, hypertension, sex differences, sRAGE, metabolically healthy

## Abstract

Males present higher blood pressure (BP) values, higher prevalence of elevated BP, and a different prevalence of cardiometabolic risk factors when compared with females. We assumed that the trends of risk markers across BP categories (normotension, high normal BP, and hypertension) differ in young males and females, and between subjects without metabolic abnormalities (without obesity, insulin resistance, atherogenic dyslipidemia, hyperuricemia, or microinflammation) and those presenting them. Data from 2543 subjects (48% males) aged from 16 to 23 years were analyzed. The findings showed that 15% of males and 4% of females presented high normal BP while 9% and 1%, respectively, had hypertension. In males, variables characterizing obesity status, insulin sensitivity, atherogenic dyslipidemia, uric acid, adiponectin, a soluble receptor for advanced glycation end-products, and leukocyte counts showed worsening trends across BP categories. Females presented significant trends only for obesity measures, LDL-cholesterol, and non-HDL-cholesterol. Across BP categories, trends of variables characterizing cardiometabolic risk differed among abnormalities-free and presenting males. The multivariate model selected measures of central obesity, atherogenic dyslipidemia, insulin resistance, and uric acid as significant predictors of BP in both genders, and C-reactive protein in females. Sex differences in measures of cardiovascular health in juveniles may remain undiscovered unless two sexes are analyzed separately. These differences may have implications for sex-specific disease risk in adulthood.

## 1. Introduction

In adults, hypertension (HT) is a leading precursor of cardiovascular disease and strongly associates with an increased risk for cardiovascular events, renal disease, and mortality [1]. Rise in blood pressure (BP) is closely linked to an increase in measures of obesity, which, in turn, associate with a decline in insulin sensitivity and worsening of an atherogenic lipid profile [2,3,4]. In addition to anthropometric measures, it is linked with factors of glucose homeostasis, a lipid profile, other metabolic markers, such as uric acid, adiponectin, and soluble receptor for advanced glycation end products (sRAGE) levels, markers of renal function, inflammation, and oxidative status associated with BP. Uric acid is considered to be a true modifying and possibly causal factor for essential hypertension, particularly in juveniles [5]. Mechanisms comprise, among others, a reduction of nitric oxide levels in the endothelium, induction of oxidative stress, and activation of the renin–angiotensin system [6]. The relationship between BP and the kidney is complex, and each may adversely affect the other. Essential hypertension is an initiating risk factor for future end-stage renal disease, even in adolescents, regardless of their sex, and presence or absence of obesity [7]. On the other hand, an increase in arterial pressure is needed to maintain salt and water balance in the presence of restriction in renal perfusion, glomerular injury, and a reduced glomerular filtration rate [8]. Adiponectin, secreted by adipocytes, plays a role in the development of obesity-associated hypertension and insulin resistance [9]. Oxidative stress may act as a trigger for both hypertension and inflammation [10]. Homocysteine may contribute to rise in BP via induction oxidative-damage and inflammatory-damage to vasculature [11]. Whether inflammation is a cause or effect of hypertension remains unclear. Since this relationship is further confounded by the fact that several factors associated with hypertension, such as obesity or insulin resistance, are also associated with inflammation [10,12]. A role of a cell surface multi-ligand pattern recognition receptor for advanced glycation end products (RAGE) in pathogenesis of hypertension cannot be excluded. RAGE-ligand interaction initiates downstream pathways, which results in the production of reactive oxygen species, atherogenic, and inflammatory responses, eventually potentiating vasoconstriction, peripheral vascular resistance, and arterial stiffness [13]. Circulating sRAGE variants possess the ligand binding domain but lack the transmembrane and the signaling domain. Thus, sRAGE may exert a protective role by reducing the pool of ligands interacting with cellular RAGE.

Elevated BP (e.g., high normal BP (HNBP) and HT) associates with adverse cardiac and vascular changes such as increased left ventricular mass, carotid thickness, arterial stiffness, decreased diastolic function, and endothelial dysfunction, which are already found in children and adolescents [14]. It is well documented that overweight-associated or obesity-associated HNBP and HT is already in juveniles accompanied with worsened markers of cardiometabolic risk, e.g., with elevated total cholesterol, low-density lipoprotein cholesterol (LDL-C), triacylglycerols, fasting plasma insulin, C-reactive protein (CRP), uric acid, reduced high-density lipoprotein cholesterol (HDL-C), adiponectin, and insulin sensitivity [3,4,15]. We showed that, in young individuals, markers of adiposity, biochemical indicators of cardiometabolic risk, and a continuous metabolic syndrome score, increase across BP categories (normotension - NT, HNBP, HT) not only in subjects presenting cardiometabolic abnormalities but even in their peers displaying a metabolically healthy phenotype, e.g., in subjects without central obesity, insulin resistance, atherogenic dyslipidemia, microinflammation, and hyperuricemia [16]. 

The prevalence of metabolic syndrome (MetSy), its components, and other cardiometabolic risk markers such as hyperuricemia or elevated C-reactive protein levels differs between males and females [17,18,19,20,21]. Until about the age of 70, males have higher BP and a higher prevalence of HT than females [22]. In our above mentioned study, about 42% of normotensive juveniles were males, Males accounted for about 81% of individuals with HNBP and 90% of hypertensive subjects [16]. In multivariate analyses, the male sex appeared to be a significant predictor of both systolic (SBP) and diastolic BP (DBP), regardless of the presence or absence of cardiometabolic abnormalities. Former Slovak studies in young subjects document that, similarly to the prevalence of elevated BP, that of other components of metabolic syndrome or hyperuricemia differs between males and females [19,23]. These lines of evidence led us to the assumption that the trends of indicators of cardiometabolic risk across BP categories differ between sexes. We hypothesized that associations would be less expressed in females since young females present lower BP values and lower prevalence of elevated BP when compared with males [19,22]. To this point, we re-analyzed the trends for traditional cardiometabolic risk markers (i.e., proxy measures of obesity, glucose homeostasis, lipid profile) as well as those of oxidative status, inflammatory markers, renal function, concentrations of uric acid, adiponectin, and sRAGE across BP categories separately in males and females. We also explored whether these trends manifest similarly in the presence or absence of cardiometabolic abnormalities.

## 2. Materials and Methods 

### 2.1. Study Design

Design of the study was described in detail previously [16,24]. Studied subjects were students of state secondary schools in the Bratislava region. The subjects voluntarily decided to participate in the “Respect for Health” survey. This aimed to gather information on cardiometabolic health of students. Exclusion criteria were any acute or chronic illness, and pregnancy or lactation in females. In the present analysis, 2543 non-diabetic White Caucasians of Central European descent (48.1% males) aged from 16 to 23 years, presenting CRP < 10 mg/L, and estimated glomerular filtration rate >1 mL/s/1.73 m^2^ were included [16]. A written informed consent from parents or caregivers of children under 18 years of age, and from the full-aged participants, was obtained. The study was approved by the Ethics Board of the Health Department of the Bratislava Self-governing Region. All the procedures were in accordance with the Declaration of Helsinki.

### 2.2. Anthropometric and Blood Pressure Measurements

Anthropometric and BP measurements were performed directly at high schools on an appointed day, in barefoot subjects wearing light clothes, by trained personnel, according to standard anthropometric protocols, as described previously [24]. Height was measured with a portable stadiometer, waist circumference using a flexible tape, body weight using digital scales (Omron BF510, Kyoto, Japan) equipped for determining total body fat percentage employing the bio-impedance method. Height and waist circumference were approximated to the nearest 0.5 cm and body weight measured to the nearest 0.1 kg. Intra-examiner and inter-examiner variation coefficients for anthropometric measurements were <3%. Body mass index (BMI), waist-to-height ratio (WHtR), and fat-free mass [25] were calculated. Three BP and heart rate measurements using an automated device (Omron M-6 COMFORT, Kyoto, Japan) were taken on the right arm, in subjects seated for 10 min. The mean of the last two readings was recorded, with intra-examiner and inter-examiner variation for BP at about 3% and 4%, respectively.

### 2.3. Biochemical Analyses

Blood was sampled from participants after overnight fasting. At the central laboratory, blood counts (Sysmex XE-2100 analyser, Sysmex Corporation, Kobe, Japan) and standard blood chemistry analyses (plasma fasting glucose, insulin, total cholesterol, HDL-C, triacylglycerols, creatinine, highly sensitive CRP, and uric acid) were performed (ADVIA analysers, Siemens, Erlangen, Germany). Plasma total L-homocysteine was measured using a fluorescence polarization immunoassay (Abbott AxSym analyzer, Abbott Park, IL, USA). Microalbuminuria was assessed in spot urine as albumin (immuno-turbidimetrically) to creatinine ratio. LDL-C concentration (Friedewald equation), non-HDL-C, atherogenic index of plasma [26], the Quantitative Insulin Sensitivity Check Index (QUICKI) [27], and estimated glomerular filtration rate (using the equation for the full age spectrum [28]) were calculated. Enzyme-linked immunoassays were employed to determine plasma concentrations of adiponectin, soluble RAGE (sRAGE, Quantikine, measuring the total pool of soluble forms of RAGE, including an endogenous secretory RAGE (esRAGE), R&D, Minneapolis, MN, USA), and esRAGE (B-Bridge International, Inc., Mountain View, CA, USA), according to the manufacturers’ instructions. The inter-assay coefficient of variation for adiponectin determination was 12.8% (*n* = 20, mean: 15.2 mg/L). For sRAGE (*n* = 31, mean: 1423 ng/L), and esRAGE (*n* = 32, mean: 348 ng/L), it equaled 9.9%. Intra-assay coefficients of variation were 8.3%, 4.8%, and 5.2%, respectively. Cleaved RAGE (cRAGE) was calculated as a difference between sRAGE and esRAGE. Concentrations of thiobarbituric acid reactive substances (TBARS, fluorometrically) [29] were measured (Sapphire II instrument, Tecan, Vienna, Austria).

### 2.4. Classification of Blood Pressure, Cardiometabolic Risk Factors, and Abnormalities

Normotension was classified as SBP < 130 mmHg and DBP < 85 mmHg, HNBP as SBP 130–139 mmHg and/or DBP 85–90 mmHg, and hypertension as SBP ≥ 140 mmHg and/or DBP ≥ 90 mmHg [30]. Since the age-specific and sex-specific waist circumference percentiles for Slovak adolescents are not available, we used WHtR as a proxy measure of central obesity. Subjects not presenting either cardiometabolic risk factors, e.g., central obesity (WHtR ≥ 0.5), elevated fasting glycemia (≥5.6 mmol/L), elevated triacylglycerols (≥1.7 mmol/L), low HDL-C (<1.03 mmol/L (males) and <1.29 mmol/L (females)), and biomarkers, e.g., elevated atherogenic index (≥0.11), elevated uric acid (≥420 μmol/L (males), ≥340 μmol/L (females)), elevated fasting insulinemia (≥20 μIU/mL), and elevated CRP (>3 mg/L) were considered as metabolic abnormalities-free. Those manifesting ≥1 risk factors or biomarkers were classified as presenting abnormalities.

A metabolic syndrome was classified as the presence of ≥3 components out of 5 (e.g., SBP ≥ 130 mmHg or DBP ≥ 85 mmHg, WHtR ≥ 0.5, plasma glucose ≥ 5.6 mmol/L, TAG (triacylglycerols) ≥ 1.7 mmol/L, HDL-C < 1.03 mmol/L (males), and <1.29 mmol/L (females)). 

Cardiometabolic risk was estimated by calculating a continuous cardiometabolic score, using a modified formula of Soldatovic et al. [31], e.g., WHtR/0.5 + glucose/5.6 + triacylglycerols/1.7 + SBP/130 - HDL-C/1.02 (males) or 1.28 (females) + insulin/20 + uric acid/420 (males) or 340 (females) + CRP/3. To ascertain that an increasing trend of the score across BP categories is not driven solely by BP, the score was alternatively calculated by omitting the SBP component from the equation.

### 2.5. Statistical Analyses

Non-normally distributed data were logarithmically transformed. Two sets of data were compared using two-sided Student’s t-test. One-way analysis of variance (ANOVA) with a post-hoc Bonferroni test to correct for multiple comparisons was used to compare the variables between BP categories in males. Due to low prevalence of HNBP and HT in females, we examined the trends employing a non-parametric (Kruskal-Wallis) test, without further post-hoc comparison of BP categories. Frequencies were compared employing the Chi-square test. Pearson correlation coefficients were calculated. Data are given as mean ± SD, those evaluated after log transformation as a geometric mean and the range between -/+ 1SD, after back-transformation of log data, or as counts and percentages. P values <0.05 were considered statistically significant. Analyses were performed using the SPSS v.16 for Windows software (SPSS Inc., Chicago, IL, USA). 

Multivariate analysis using the Orthogonal Projections to Latent Structures (OPLS, Simca v.15 software, Sartorius Stedim Data Analytics AB, Umea, Sweden) was employed to identify the set of explanatory variables predicting the dependent ones, e.g., SBP and DBP. Waist-to-height ratio, atherogenic index, non-HDL-C, QUICKI, uric acid, CRP, leukocyte count, adiponectin, sRAGE, TBARS, and homocysteine were entered as independent variables. Alternatively, the score was calculated entering the continuous cardiometabolic score without the SBP component, leukocyte count, adiponectin, sRAGE, TBARS, and homocysteine as independent predictors. Prior to modelling, variables with high skewness and low min/max ratio were logarithmically transformed and all data were mean-centered. Variables with Variable of Importance for the Projection values ≥1.00 were considered as important contributors.

### 2.6. Sample Size Estimation

The sample size was estimated on the basis of participant to item ratio, or a minimum total sample size required for multiple regression analysis [32]. According to different suggestions, the subject to variable ratio ranges from 10:1 to 30:1. Total sample size might be roughly evaluated when employing the following scale: 50—very poor; 100—poor; 200—fair; 300—good; 500—very good; ≥1000—excellent. In multiple regression models, we tested the impact of 11 independent variables, and, in both sexes, more than 1000 subjects were analyzed. Thus, our study fulfills even the most conservative requirement on subjects to variable ratio and is in line with the “excellent” criterion.

## 3. Results

### 3.1. Between-Sex Comparison

Cohort characteristics are given in Table 1. Males presented higher mean BP values, and about 5.4-fold higher prevalence of elevated BP (HNBP + HT) when compared with females. Males also displayed higher anthropometric measures (except for the percentage of total body fat), higher glycemia, atherogenic index, continuous cardiometabolic score, uricemia, sRAGE and cRAGE, and homocysteine levels. Mean age, insulin sensitivity, triacylglycerols, esRAGE, and TBARS concentrations did not differ significantly between sexes. Other variables were higher in females. The prevalence of elevated insulinemia and triacylglycerolemia did not differ significantly between sexes. That of low HDL-C and elevated CRP was higher among females. The remaining risk markers were more frequent among males. 

### 3.2. Males

About 73% of males were normotensive, 18% presented HNBP, and 9% were hypertensive (Table 2). Among all males, the prevalence of elevated SBP reached 26%, and that of elevated DBP reached 7%. While the prevalence of elevated SBP did not differ significantly between males with HNBP and HT, that of elevated DBP was higher among HT compared to HNBP presenting subjects (Table 2). SBP, DBP, heart rate, anthropometric measures, and the prevalence of central obesity displayed significant increasing trends across BP categories. Glucose concentrations and the prevalence of elevated glycemia were similar across BP categories. Insulin levels displayed an increasing trend across BP categories (without a significant rise in the prevalence of elevated fasting insulin), which resulted in a declining trend of insulin sensitivity. In comparison with NT subjects, males with HNBP and hypertension displayed higher cholesterol, LDL-C, non-HDL-C, and triacylglycerols, but not HDL-C concentrations. Atherogenic index showed an increasing trend, but the post-hoc test did not indicate a significance between categories. The prevalence of low HDL-C, elevated triacylglycerols, or atherogenic index did not differ significantly between BP categories. Continuous cardiometabolic score (even if calculated without the SBP component) as well as the prevalence of MetSy increased across the BP categories. A continuous cardiometabolic score calculated without the SBP component correlated significantly with SBP (y = 0.0114x + 1.3617, r = 0.142, *p* < 0.001). Significant differences between BP categories were also observed for uric acid (with increasing prevalence of elevated levels across BP groups), sRAGE, esRAGE, and cRAGE concentrations, and leukocyte counts. ANOVA also indicated significant trend for adiponectin, but the post-hoc test failed to reveal significant between-categories differences. Variables characterizing renal function, CRP, TBARS, and homocysteine concentrations did not differ significantly between BP categories.

The OPLS model selected waist-to-height ratio, QUICKI, atherogenic index of plasma, and non-HDL-C as significant determinants of both SBP and DBP, while SBP was also associated significantly with uric acid. The models poorly explained variability in BP (Table 3). The alternative model indicated in both cases a significant impact of the continuous cardiometabolic score, and that of sRAGE, with R^2^ even lower than in the previous model (data not presented). 

#### 3.2.1. Males Presenting Cardiometabolic Abnormalities

About 43% of males manifested at least one cardiometabolic abnormality. Among them, 66% were normotensive, 22% presented HNBP, and 12% presented hypertension (Table 4). Furthermore, 32% of males presenting abnormalities suffered from elevated SBP. The prevalence of elevated DBP reached 9%. The prevalence of elevated SBP was similar among subjects with HNBP and HT, while that of elevated DBP was more frequent in subjects with HT. Mean SBP, DBP, heart rate, anthropometric variables, insulinemia, QUICKI, total cholesterol, LDL-C, non-HDL-C, continuous cardiometabolic score, and uric acid showed significant trends across BP categories. The prevalence of central obesity and MetSy increased across BP categories (Table 4).

#### 3.2.2. Males Not Presenting Cardiometabolic Abnormalities

The prevalence of NT, HNBP, and HT (78%, 15%, and 7%, respectively), as well as that of elevated SBP and elevated DBP (Table 5) differed significantly from the prevalence observed in males presenting abnormalities (Table 4). Comparison of males presenting and not presenting cardiometabolic abnormalities revealed that two groups differ significantly in all variables except for age, height, renal function markers, sRAGE, TBARS, and homocysteine concentrations (Table 5). 

Similarly to males presenting cardiometabolic abnormalities, SBP, DBP, and heart rate showed increasing trends across BP categories (Table 5). Abnormalities presenting males (Table 4) and abnormalities-free males (Table 5) displayed similar mean BP values in corresponding BP categories. 

In abnormalities-free males, anthropometric measures showed significant trends across BP categories. Except for height, anthropometric measures differed significantly from those displayed by males presenting abnormalities. Insulinemia, QUICKI, non-HDL-C, and uric acid concentrations showed significant trends across BP categories and at least in some BP categories, which differed significantly from the values observed in males with abnormalities. A continuous cardiometabolic score, even after the exclusion of the SBP component, remained significant across BP categories and was significantly lower in all BP categories when compared with abnormalities presenting males. As in males presenting abnormalities, glycemia, HDL-C, triacylglycerols, atherogenic index, CRP, and leukocyte counts did not show significant trends across BP categories, but, in some BP categories, their mean values differed significantly from those presented by males with abnormalities. Microalbuminuria, adiponectin, TBARS, and homocysteine concentrations neither differed significantly across BP categories, nor from levels in corresponding BP categories in abnormalities presenting males. In contrast with males presenting abnormalities, no significant trend in total-cholesterol and LDL-cholesterol concentrations across BP categories had been revealed in abnormalities-free subjects. Moreover, all sRAGE variants showed decreasing concentrations across BP categories.

### 3.3. Females

Of 1321 females, about 2% presented elevated SBP and about 4% presented elevated DBP (Table 1). Thus, 95% of females were normotensive, about 4% displayed HNBP, and about 1% presented HT (Table 6). The prevalence of elevated SBP or DBP did not differ significantly between females presenting HNBP and HT.

Anthropometric variables (except for height) displayed worsening trends across BP categories, and the prevalence of central obesity was higher in females presenting HT when compared with those displaying HNBP (Table 6). Total cholesterol, LDL-C, non-HDL-C concentrations, continuous cardiometabolic score (even after omission of SBP component), and the prevalence of elevated triacylglycerols and that of MetSy displayed significant increasing trends across BP categories. A continuous cardiometabolic score calculated without the SBP component correlated significantly with SBP (y = 0.0144x + 1.1735, r = 0.149, *p* < 0.001). 

The OPLS model selected waist-to-height ratio, QUICKI, atherogenic index of plasma, non-HDL-C, uric acid, and CRP as independent predictors of both SBP and DBP. The models poorly explained the variability in SBP or DBP (Table 3). In the alternative model, a continuous cardiometabolic score and leukocyte counts predicted both SBP and DBP, but the variability explained by these models was lower when compared with the previous one (data not presented). 

Low prevalence of elevated BP among females did not allow for plausible evaluation of the trends across BP categories separately in abnormalities presenting females and abnormalities-free females. Data are given in Supplementary Materials Tables S1 and S2, respectively. Additionally, 558 (42%) of females presented cardiometabolic abnormalities. Among them, 5% had HNBP, and 1% presented HT. About 4% of abnormalities-free females manifested HNBP and <1% had hypertension.

## 4. Discussion

Representative data on the prevalence of HNBP and hypertension in Slovak adolescents or young adults are not available. Among medical students aged 23 years in mean, 18% of males and 2% of females presented hypertension [33]. A study in students aged from 18 to 20 years indicated 41% prevalence of HNBP and 30% of hypertension in males, and 18% and 16%, respectively, in females [34]. The Slovak Health Advice Centers reported about 32% prevalence of elevated BP in males aged from 10 to 25 years, and 12% among females [19]. Recent European studies in juveniles indicated 10–16% prevalence of prehypertension (a former term for HNBP) and a 13–44% prevalence of hypertension in males, and 11–13% and 6–21%, respectively, in females [35,36,37]. Comparison of the prevalence rates is cumbersome due to age-differences of studied cohorts and particularly due to methodological differences in classification of elevated BP. Two Slovak studies used the same methodology as we did. However, the study among students was small (*n* = 122, 18% males) [34] while the other one reported on a specific population, which involved individuals actively seeking health counseling provided by The Health Advice Centers [19]. The study in medical students classified BP as optimal, normal, and elevated [33]. Thus, it did not report data on the prevalence of HNBP. Other studies [35,36,37] classified BP status according to the 2004 Working Group normative, e.g., according to sex-specific, age-specific, and height-specific percentile charts [38]. Despite these differences, the prevalence of elevated BP was consistently higher among males when compared with females. On the other hand, in our study and the other Slovak and the Italian studies [34,35], the prevalence of HNBP/prehypertension exceeded that of hypertension. However, in Portuguese and Lithuanian juveniles, the prevalence of HT was higher than that of prehypertension [36,37]. Except for the prevalence of HNBP in our males, which was roughly similar to that reported in other studies, the prevalence of hypertension in males and both as well as HNBP and hypertension in females was lower in our study [35,36,37]. Reasons are unclear. A bias imposed by voluntary participation cannot be excluded. Data on individuals who did not take part in our survey are not available. Thus, generalizability of our results to populations not represented herein remains unknown.

In our study, the majority of assessed variables differed significantly between males and females. Sex differences in anthropometric measures, HDL-C, uric acid, adiponectin concentrations, or microalbuminuria reflect biological variability, which is translated into different reference ranges for males and females. Even for variables that do not have sex-specific reference ranges, sex differences (within the reference range) are well documented. Therefore, premenopausal females present lower BP values when compared to males of similar age, higher insulin sensitivity, less proatherogenic plasma lipid profile, and higher CRP concentrations, leukocyte counts, adiponectinemia, sRAGE, and lower homocysteine concentrations [22,39,40,41,42,43,44,45,46]. Of interest, we revealed no sex difference in insulin sensitivity, despite differences in glycemia and insulinemia. We suppose that statistically significant sex-differences in glucose and insulin concentrations, or estimated glomerular filtration rate, which manifested within their reference ranges, do not imply clinical significance and rather reflect the effects of large sets of data. Studies in a general population of older adults reported higher sRAGE concentrations in females, by about 16–25% [45,46]. Thus, observed statistical significance between the sex difference of about 3% in sRAGE and cRAGE concentration should also be considered clinically insignificant. To our knowledge, data on sex-differences in esRAGE and cRAGE in an apparently healthy population are not available.

Sex-differences in measures of cardiovascular health observed in adults may not manifest in young subjects. Thus, we aimed to compare our data with those from other studies on individuals of similar age to our subjects. Consistently with our data, other studies show that females display lower SBP [47,48,49], higher body fat percentage, lower lean mass [47,48], lower glycemia, higher insulinemia [48,50], higher HDL-C levels [47,48,51], and higher CRP concentrations [52] when compared with males. Data on sex differences in DBP, BMI, total cholesterol, LDL-C, and triacylglycerols are inconsistent [47,48,49,51]. In contrast to other studies [47,48,51], we revealed no sex differences in triacylglycerolemia, and our females showed higher concentrations of all types of cholesterol when compared with males. Lacking a difference in triacylglycerol levels might reflect a low prevalence of hypertriacylglycerolemia, which is also observed in other Slovak studies [18,19]. However, as expected, our females presented a less proatherogenic lipid profile compared with males, and less severe continuous cardiometabolic score. A small study in young adults reported only a tendency towards higher sRAGE concentrations in females [53]. Thus, regarding sex differences, our population showed only minor deviations from other data.

Changes in means may not be sensitive enough to detect variations occurring at the extremities of the distribution. These are captured by trends. Across BP categories, trends of variables associated with cardiometabolic risk differed between the sexes. Trends observed in males were largely consistent with those obtained if both sexes were analyzed together [16]. In contrast to the whole cohort, we did not observe a significant trend in glycemia, HDL-cholesterol, microalbuminuria, and homocysteine concentrations either in males or in females. However, in both sexes, a significant upward trend in total cholesterol, and, in males, an upward trend in leukocyte counts, was revealed. [16] Contrary to findings in the whole cohort and in males, trends in insulinemia, QUICKI, triacylglycerols, atherogenic index, uricemia, adiponectin, sRAGE, cRAGE, and esRAGE across BP categories were insignificant in females. Hence, in males, variables characterizing obesity status, insulin sensitivity, atherogenic dyslipidemia, concentrations of uric acid, adiponectin, sRAGEs, and leukocyte counts showed worsening trends across BP categories while females presented significant trends only for obesity measures, LDL-C, and non-HDL-cholesterol. These data seem to support the view that young females have more favorable risk profiles when compared with their male counterparts, not only in terms of BP but also in terms of cardiovascular risk factors and markers [54].

The different outcomes in trends might have been influenced by the low prevalence of elevated BP in our females. This assumption is supported by the fact that multivariate regression models indicated that predictors of BP show only slight sex-differences. Even variables that do not display significant trends across BP categories in females (i.e., insulin resistance, atherogenic index of plasma, non-HDL-C, and uric acid) are associated significantly with higher BP in both sexes. This finding is in line with a well-known fact that hypertension frequently presents concurrently with other cardiovascular disease risk factors, such as central obesity, insulin resistance, and atherogenic dyslipidemia, constituting the MetSy [55,56,57]. These factors individually and synergistically influence the pathophysiology of hypertension. The causes and mechanisms of the MetSy are diverse but clustering of the components in MetSy confers higher probability of manifestation of cardiovascular and renal diseases, diabetes, and mortality when compared with the manifestation of isolated components [55,57]. However, the increased risk imposed by the MetSy may vary by the absence or presence of hypertension. This is reflected by lower values of a continuous cardiometabolic score calculated excluding the SBP component from the equation. A higher continuous metabolic score indicates a less favorable cardiometabolic profile. 

Hypertension often occurs simultaneously with other markers of increased cardiometabolic risk, such as hyperuricemia, and low-grade inflammation, which are not part of any definition of MetSy. Uric acid, which is the end-product of purines metabolism in humans, is considered to play a role in pathogenesis of essential hypertension in juveniles [5]. It may induce insulin resistance and higher uric acid levels increase the risk of later development of atherogenic dyslipidemia [58,59]. Except for BP, uricemia also correlates with measures of obesity, glucose homeostasis, lipid profile, and inflammatory markers [60,61,62,63]. The C-reactive protein, which is a non-specific marker of an inflammatory reaction, is a significant cardiovascular risk factor [64]. CRP correlates with all components of MetSy as well as with uricemia even in children and adolescents [52,65,66]. A significant impact of CRP in our females suggests that elevated BP-associated low-grade inflammation might be sex-specific, at least in young subjects. This is in line with the finding that CRP is associated with MetSy in females but not in males [67]. However, a tested panel of independent variables poorly explained variability in BP, which indicated that other factors not assessed in our study, e.g., genetic background, different biochemical markers, family history, environmental and behavioral factors, such as smoking, alcohol consumption, diet, physical activity, sedentary behavior, socioeconomic status, etc., might be more robust determinants of BP. Moreover, sex-differences in BP are at least partially attributable to sex hormones and their receptors [54].

About 47% of our males and 50% of females with elevated BP were cardiometabolic abnormalities-free, i.e., insulin sensitive, not presenting central obesity, atherogenic dyslipidemia, CRP > 3 mg/L, or hyperuricemia. Thus, the presence of cardiometabolic abnormalities seemed not to be a prerequisite for manifestation of HNBP or HT. Equations approximating the relationship between a continuous cardiometabolic score (calculated without the SBP component) and SBP in our study indicate that the same increase in SBP would be associated with a higher increase in risk score in females when compared with males, and that, at a given SBP value, the score is higher in females. Clinical relevance of this finding might be questioned, as premenopausal females generally present lower BP values when compared with males. However, these models indicate that a normotensive male and a normotensive female presenting SBP equal to the mean value observed for their sex in our study would score equally (i.e., 2.70). In hypertensive subjects, the cardiometabolic score would be similar (3.03 in males and 3.05 in females). Females displaying SBP equal to values presented by our males would score 2.85 and 3.28, respectively. These data raise a question whether the presence of hypertension imposes higher cardiometabolic risk in young females when compared with males.

In males, continuous cardiometabolic score increased across BP categories both in cardiometabolic abnormalities-presenting (by about 13%) and abnormalities-free subjects (about 14%). This rise was not solely on the account of increased BP. After exclusion of the SBP component, abnormalities-presenting hypertensive males displayed about a 10% higher score than their normotensive peers while, in abnormalities-free males, the difference reached about 9%. Thus, in apparently healthy young subjects with elevated BP, clinicians should pay attention even to a rise of risk factors and markers occurring within the “normal range”.

In contrast to our hypothesis, males in corresponding BP categories displayed similar mean BP values regardless of the presence or absence of cardiometabolic abnormalities. As expected, those manifesting abnormalities displayed less favorable values of almost all variables of cardiometabolic risk when compared with their abnormalities-free counterparts. Variables generally presented worsening trends across BP categories. Of interest, cardiometabolic abnormalities-free males maintained similar adiponectinemia across BP categories despite increasing trends in measures of obesity and insulin resistance. This finding is inconsistent with the data of Brambilla et al. [68], which show that non-obese hypertensive juveniles present lower adiponectin concentrations when compared to their obese, hypertensive counterparts. Moreover, in their study, adiponectin was independently related to hypertension in the adjusted multiple logistic regression model. We observed a decreasing trend in both sRAGE variants across BP categories only in cardiometabolic abnormalities-free males. Hypertensive adults present lower sRAGE levels when compared with their normotensive counterparts and an inverse association between BP and sRAGE levels [69,70]. However, virtually all components of MetSy show a significant relationship with sRAGE even in adolescents and young adults [53,71,72]. Thus, circulating RAGE levels decline with increasing BP concurrently with worsening of other cardiometabolic risk factors and biomarkers, even before these reach the threshold risk values. In the presence of cardiometabolic abnormalities, a decline in sRAGE does not seem to be independently impacted by the rise in BP. Interaction of circulating RAGEs with RAGE ligands may ameliorate tissue injury resulting from oxidative stress generation, inflammatory, atherogenic, and diabetogenic responses [73,74]. An elevated BP associated decline in sRAGE in abnormalities-free males warrants attention since low sRAGE levels indicate an increased risk of diabetes development, cardiovascular disease, or death in non-diabetic subjects [75].

The strengths of our study comprise a large cohort of adolescents and young adults, representing a particularly suitable group for studies of sex differences in variables characterizing cardiometabolic risk due to a low interference of comorbidities potentially affecting these targets. The study strengths also include the ability to examine a range of measures of cardiometabolic health and the use of a multivariate model suitable for evaluating large sets of data, particularly not normally distributed, and partially correlated. Our study is limited by its cross-sectional design, which allows only for comments on associations. All variables were measured at a single occasion. The generalizability of our findings to the wider population may be limited. Other limitations are mentioned throughout the discussion.

## 5. Conclusions

Sex differences in measures of cardiovascular health in their trends across BP categories as well as in predictors of BP are apparent in young subjects. These differences may remain undiscovered unless males and females are analyzed separately and may have implications for sex-specific disease risk in later life. Further studies are needed to elucidate the specific mechanisms underlying these sex differences and how sex differences track into adulthood.

## Figures and Tables

**Table 1:** Cohort characteristics.

**Variable**	**All**	**Males**	**Females**	***p***
*n*	2543	1222 (48.1%)	1 321 (51.9%)	-
Age, years	17.5 ± 1.2	17.4 ± 1.2	17.5 ± 1.2	0.119
Systolic Blood Pressure (BP), mmHg	115 ± 13	123 ± 12	107 ± 9	<0.001
Diastolic BP, mmHg	72 ± 8	73 ± 8	70 ± 8	<0.001
Heart rate, beats/min	79 ± 13	78 ± 13	81 ± 12	<0.001
Height, cm	172.1 ± 9.4	179.1 ± 6.8	165.6 ± 6.2	<0.001
Weight, kg	66.8 ± 14.0	74.1 ± 13.7	60.1 ± 10.5	<0.001
Waist, cm	75.4 ± 9.3	79.4 ± 9.0	71.6 ± 7.9	<0.001
Body Mass Index (BMI), kg/m^2^	22.5 ± 3.7	23.1 ± 3.8	21.9 ± 3.5	<0.001
Waist/height	0.44 ± 0.05	0.44 ± 0.05	0.43 ± 0.05	<0.001
Total body fat, %	24.2 ± 9.5	17.5 ± 7.2	30.3 ± 6.9	<0.001
Fat-free mass, kg	48.5 ± 11.3	58.5 ± 7.0	39.2 ± 4.6	<0.001
Glucose, mmol/L	4.8 ± 0.4	4.9 ± 0.4	4.7 ± 0.4	<0.001
Insulin, μIU/mL	9.7 (5.9; 15.9)	9.5 (5.6; 15.9)	9.9 (6.2; 15.8)	0.021
QUICKI	0.344 ± 0.026	0.344 ± 0.027	0.344 ± 0.026	0.978
Cholesterol, mmol/L	4.05 ± 0.76	3.83 ± 0.71	4.26 ± 0.76	<0.001
HDL-C, mmol/L	1.39 ± 0.30	1.25 ± 0.23	1.52 ± 0.30	<0.001
LDL-C, mmol/L	2.26 ± 0.71	2.18 ± 0.59	2.34 ± 0.61	<0.001
Non-HDL-C, mmol/L	2.66 ± 0.69	2.58 ± 0.69	2.74 ± 0.67	<0.001
Triacylglycerols, mmol/L	0.82 (0.54; 1.24)	0.79 (0.52; 1.22)	0.81 (0.54; 1.22)	0.216
Atherogenic index	−0.23 ± 0.22	−0.19 ± 0.23	−0.26 ± 0.20	<0.001
Continuous Met. score	3.62 ± 0.96	3.70 ± 1.00	3.54 ± 0.91	<0.001
Met score without SBP	2.74 ± 0.94	2.76 ± 0.99	2.72 ± 0.90	0.248
Uric acid, μmol/L	304 ± 74	354 ± 60	257 ± 51	<0.001
eGFR, mL /s/1.73 m^2^	1.77 ± 0.23	1.74 ± 0.22	1.80 ± 0.23	<0.001
U-alb/crea, mg/mmol	0.4 (0.2; 1.1)	0.4 (0.2; 0.9)	0.5 (0.2; 1.3)	<0.001
CRP, mg/L	0.5 (0.1; 1.7)	0.5 (0.1; 1.5)	0.5 (0.2; 1.8)	0.001
Adiponectin, mg/L	15.2 (7.4; 31.1)	12.3 (6.2; 24.4)	18.0 (8.9; 36.3)	<0.001
sRAGE, ng/L	1578 (1125; 2213)	1584 (1132; 2218)	1542 (1098; 2166)	0.045
esRAGE, ng/L	327 (215; 500)	313 (204; 480)	311 (205; 472)	0.818
cRAGE, ng/L	1236 (861; 1773)	1254 (862; 1824)	1218 (857; 1731)	0.044
TBARs μmol/L	1.32 (0.74; 2.35)	1.32 (0.75; 2.34)	1.31 (0.72; 2.36)	0.616
Homocysteine, μmol/L	8.4 (6.0; 11.9)	11.3 (7.8; 16.4)	9.5 (7.1; 12.8)	<0.001
Leukocytes, 10^9^/L	6.6 ± 1.6	6.3 ± 1.4	6.8 ± 1.8	<0.001
**Prevalence**				***p*** **_Chi_**
**Elevated**				
Systolic BP, *n* (%)	334 (13.1)	314 (25.7)	20 (1.5)	<0.001
Diastolic BP, *n* (%)	140 (5.5)	83 (6.8)	57 (4.3)	0.006
Waist/height, *n* (%)	288 (11.3)	162 (13.3)	126 (9.5)	0.003
Glucose, *n* (%)	107 (4.2)	80 (6.5)	27 (2.0)	<0.001
Insulin, *n* (%)	177 (7.0)	96 (7.9)	81 (6.1)	0.088
Triacylglycerols, *n* (%)	133 (5.2)	68 (5.6)	65 (4.9)	0.466
Atherogenic index, *n* (%)	158 (6.2)	108 (8.8)	50 (3.8)	<0.001
Uric acid, *n* (%)	234 (9.2)	164 (13.4)	70 (5.3)	<0.001
CRP, *n* (%)	225 (8.8)	86 (7.0)	139 (10.5)	0.002
Low HDL-C, *n* (%)	480 (18.9)	176 (14.4)	304 (23.0)	<0.001
Metabolic Sy., *n* (%)	69 (2.7)	50 (4.1)	19 (1.4)	<0.001

BP—blood pressure. BMI—body mass index. QUICKI—quantitative insulin sensitivity check index. HDL-C—high- density lipoprotein cholesterol. LDL-C—low-density lipoprotein cholesterol. Met—metabolic. eGFR—estimated glomerular filtration rate. U-alb/crea—urinary albumin-to-creatinine ratio. CRP—C-reactive protein. sRAGE—soluble receptor for advanced glycation end products. esRAGE—endogenous secretory RAGE. cRAGE—cleaved RAGE. TBARs—thiobarbituric acid reactive substances. Sy: syndrome. Chi: chi-square, data are presented as mean ± SD, or as geometric mean (−/+ 1SD range) of the back-transformed log data, or as counts (percentage). Data not fitting to normal distribution were logarithmically transformed prior to statistical analysis.

**Table 2:** Characteristics of males.

**Variable**	**Normotensive**	**High Normal Blood Pressure (BP)**	**Hypertensive**	***p*** **_ANOVA_**
*n*	891	220	111	
Age, years	17.4 ± 1.2	17.6 ± 1.2	17.8 ± 1.2 **	0.001
Systolic BP, mmHg	117 ± 8	133 ± 4 ***	146 ± 8 ***^,+++^	<0.001
Diastolic BP, mmHg	70 ± 6	77 ± 6 ***	83 ± 9 ***^,+++^	<0.001
Heart rate, beats/min	77 ± 12	77 ± 14	84 ± 14 ***^,+++^	<0.001
Height, cm	178.5 ± 6.8	179.9 ± 6.1 *	182.0 ± 6.8 ***^,+^	<0.001
Weight, kg	71.4 ± 12.3	78.9 ± 13.4 ***	86.1 ± 16.2 ***^,+++^	<0.001
Waist, cm	78.0 ± 8.2	81.7 ± 8.9 ***	86.4 ± 11.1 ***^,+++^	<0.001
BMI, kg/m^2^	22.4 ± 3.5	24.4 ± 3.7 ***	26.0 ± 4.6 ***^,+++^	<0.001
Waist/height	0.44 ± 0.05	0.45 ± 0.05 ***	0.48 ± 0.06 ***^,+++^	<0.001
Total body fat, %	16.4 ± 6.8	19.4 ± 6.9 ***	22.2 ± 7.8 ***^,++^	<0.001
Fat-free mass, kg	57.2 ± 6.5	60.9 ± 6.4 ***	64.5 ± 7.5 ***^,+++^	<0.001
Glucose, mmol/L	4.9 ± 0.4	5.0 ± 0.4	5.0 ± 0.4	0.148
Insulin, μIU/mL	9.0 (8.8; 9.1)	10.6 (6.3; 17.8) ***	11.4 (6.8; 19.0) ***	<0.001
QUICKI	0.347 ± 0.028	0.338 ± 0.027 ***	0.334 ± 0.025 ***	<0.001
Cholesterol, mmol/L	3.78 ± 0.68	4.00 ± 0.74 ***	3.98 ± 0.82 **	<0.001
HDL-C, mmol/L	1.25 ± 0.23	1.25 ± 0.22	1.24 ± 0.23	0.906
LDL-C, mmol/L	2.14 ± 0.58	2.33 ± 0.60 ***	2.28 ± 0.66 *	<0.001
Non-HDL-C, mmol/L	2.53 ± 0.66	2.75 ± 0.72 ***	2.74 ± 0.81 **	<0.001
Triacylglycerols, mmol/L	0.78 (0.51; 1.19)	0.82 (0.52; 1.29)	0.87 (0.56; 1.37) *	0.016
Atherogenic index	−0.20 ± 0.23	−0.18 ± 0.22	−0.15 ± 0.23	0.043 ^a^
Continuous Met score	3.58 ± 0.94	3.92 ± 1.04 ***	4.24 ± 1.17 ***^,+^	<0.001
Met score without SBP	2.68 ± 0.94	2.90 ± 1.04 *	3.12 ± 1.17 ***	<0.001
Uric acid, μmol/L	350 ± 60	359 ± 60	377 ± 59 ***^,+^	<0.001
eGFR, mL /s/1.73 m^2^	1.75 ± 0.23	1.73 ± 0.23	1.72 ± 0.20	0.444
U-alb/crea, mg/mmol	0.4 (0.2; 1.0)	0.4 (0.2; 0.8)	0.3 (0.2; 0.8)	0.097
CRP, mg/L	0.4 (0.1; 1.4)	0.5 (0.1; 1.5)	0.6 (0.2; 1.8)	0.069
Adiponectin, mg/L	13.2 (6.7; 26.1)	11.9 (6.0; 23.5)	11.4 (5.6; 23.4)	0.028 ^a^
sRAGE, ng/L	1612 (1151; 2256)	1537 (1089; 2171)	1463 (1077; 1987) *	0.006
esRAGE, ng/L	319 (209; 487)	305 (196; 475)	324 (211; 498) **	0.007
cRAGE, ng/L	1280 (907; 1806)	1195 (727; 1965) *	1170 (856; 1599) *	0.007
TBARs μmol/L	1.30 (0.73; 2.32)	1.36 (0.80; 2.30)	1.48 (0.87; 2.51)	0.076
Homocysteine, μmol/L	11.3 (7.7; 16.4)	11.2 (7.8; 16.0)	11.6 (7.9; 17.0)	0.701
Leukocytes, 10^9^/L	6.3 ± 1.4	6.3 ± 1.3	6.7 ± 1.5 *	0.038
**Prevalence**				***p_Chi_***
**Elevated**				
Systolic BP, *n* (%)	-	206 (93.6)	108 (97.3)	0.154
Diastolic BP, *n* (%)	-	35 (15.9)	48 (43.2)	<0.001
Waist/height, *n* (%)	87 (9.8)	41 (18.6)	34 (30.6)	<0.001
Glucose, *n* (%)	53 (5.9)	18 (8.2)	9 (8.1)	0.382
Insulin, *n* (%)	62 (7.0)	21 (9.5)	13 (11.7)	0.126
Triacylglycerols, *n* (%)	46 (5.2)	14 (6.4)	8 (7.2)	0.574
Atherogenic index, *n* (%)	73 (8.2)	22 (10.0)	13 (11.7)	0.374
Uric acid, *n* (%)	105 (11.8)	35 (15.9)	24 (21.6)	0.008
CRP, *n* (%)	61 (6.8)	16 (7.3)	9 (8.1)	0.877
Low HDL-C, *n* (%)	127 (14.3)	31 (14.1)	18 (16.2)	0.848
Metabolic syndrome, *n* (%)	12 (1.3)	20 (9.1)	18 (16.2)	<0.001

BP—blood pressure. ANOVA: analysis of variance. BMI—body mass index. QUICKI—quantitative insulin sensitivity check index. HDL-C—high- density lipoprotein cholesterol. LDL-C—low-density lipoprotein cholesterol. Met—metabolic. eGFR—estimated glomerular filtration rate. U-alb/crea—urinary albumin-to-creatinine ratio. CRP—C-reactive protein. sRAGE—soluble receptor for advanced glycation end products. esRAGE—endogenous secretory RAGE. cRAGE—cleaved RAGE. TBARs—thiobarbituric acid reactive substances. Chi: chi-square, data are presented as mean ± SD, data not fitting to normal distribution were logarithmically transformed prior to statistical analysis and are given as geometric mean (−1SD; +1SD range) of the back-transformed log data, or as counts (percentage). ^a^: The post-hoc tests did not localize the between-group difference. *, **, ***: *p* < 0.05, 0.01, 0.001, respectively, vs. normotensive subjects. ^+^, ^++^, ^+++^: *p* < 0.05, 0.01, 0.001, vs. subjects with high normal blood pressure

**Table 3:** Multivariate regression on systolic and diastolic blood pressure using the Orthogonal Projections to Latent Structures model in males and females.

Variable	Males		Females	
	Systolic BP	Diastolic BP	Systolic BP	Diastolic BP
Waist/height	1.73	1.55	1.88	1.52
QUICKI	1.04	1.32	1.15	1.33
Atherogenic index	1.17	1.27	1.12	1.19
Non-HDL-C	1.18	1.25	1.12	1.17
Uric acid	1.02	0.87	1.01	1.06
CRP	0.87	0.91	1.05	1.02
Leukocytes	0.63	0.69	0.83	0.80
Adiponectin	0.70	0.44	0.54	0.56
sRAGE	0.97	0.63	0.56	0.71
TBARS	0.68	0.82	0.17	0.52
Homocysteine	0.19	0.60	0.42	0.48
R^2^	0.11	0.09	0.07	0.06

BP—blood pressure. QUICKI—quantitative insulin sensitivity check index. HDL-C—high- density lipoprotein cholesterol. CRP—C-reactive protein. sRAGE—soluble receptor for advanced glycation end products. TBARS—thiobarbituric acid reactive substances. Variables with Variable of Importance for the Projection values ≥1.00 were considered as important contributors.

**Table 4:** Characteristics of males presenting cardiometabolic risk factors.

**Variable**	**All**	**Normotensive**	**High Normal BP**	**Hypertensive**	***p_ANOVA_***
*n*	525	350	114	61	
Age, years	17.5 ± 1.3	17.4 ± 1.3	17.7 ± 1.2	17.9 ± 1.4 *	0.013
Systolic BP, mmHg	124 ± 12	117 ± 8	133 ± 4 ***	145 ± 8 ***^,+++^	<0.001
Diastolic BP, mmHg	74 ± 8	71 ± 6	78 ± 6 ***	84 ± 9 ***^,+++^	<0.001
Heart rate, beats/min	79 ± 13	78 ± 13	79 ± 15	83 ± 13 **	0.007
Height, cm	179.1 ± 6.9	178.5 ± 7.0	179.5 ± 6.1	181.6 ± 7.3 **	0.004
Weight, kg	79.8 ± 16.0	76.4 ± 14.5	83.6 ± 15.2 ***	92.6 ± 17.6 ***^,++^	<0.001
Waist, cm	83.9 ± 10.7	82.0 ± 10.1	85.4 ± 10.0 **	91.9 ± 11.5 ***^,+++^	<0.001
BMI, kg/m^2^	24.9 ± 4.5	24.0 ± 4.2	25.9 ± 4.1 ***	28.1 ± 4.9 ***^,++^	<0.001
Waist/height	0.47 ± 0.06	0.46 ± 0.06	0.48 ± 0.05 *	0.51 ± 0.07 ***^,++^	<0.001
Total body fat, %	20.8 ± 8.1	19.4 ± 8.0	22.0 ± 7.4 **	26.2 ± 7.6 ***^,++^	<0.001
Fat-free mass, kg	60.9 ± 7.7	59.3 ± 7.2	62.8 ± 7.1 ***	66.9 ± 8.1 ***^,++^	<0.001
Glucose, mmol/L	5.0 ± 0.5	5.0 ± 0.4	5.0 ± 0.5	4.9 ± 0.5	0.482
Insulin, μIU/mL	11.7 (6.4; 21.4)	11.0 (5.9; 20.6)	12.8 (7.3; 22.6) *	13.2 (7.6; 23.1) *	0.015
QUICKI	0.334 ± 0.030	0.336 ± 0.032	0.328 ± 0.028 *	0.328 ± 0.027 *	0.013
Cholesterol, mmol/L	3.91 ± 0.81	3.82 ± 0.78	4.09 ± 0.83 **	4.10 ± 0.91 *	0.001
HDL-C, mmol/L	1.16 ± 0.23	1.15 ± 0.24	1.18 ± 0.22	1.16 ± 0.22	0.380
LDL-C, mmol/L	2.28 ± 0.66	2.20 ± 0.65	2.44 ± 0.64 **	2.40 ± 0.69	0.001
Non-HDL-C, mmol/L	2.75 ± 0.79	2.67 ± 0.77	2.91 ± 0.79 **	2.94 ± 0.88 *	0.004
Triacylglycerols, mmol/L	0.92 (0.56; 1.52)	0.91 (0.56; 1.51)	0.91 (0.55; 1.50)	1.00 (0.62; 1.62)	0.381
Atherogenic index	−0.09 ± 0.26	−0.09 ± 0.26	−0.11 ± 0.24	−0.06 ± 0.24	0.458
Continuous Met score	4.33 ± 1.16	4.23 ± 1.12	4.39 ± 1.18	4.78 ± 1.29 **^,+^	0.004
Met score without SBP component	3.38 ± 1.15	3.33 ± 1.11	3.37 ± 1.18	3.66 ± 1.28	0.136
Uric acid, μmol/L	379 ± 68	375 ± 69	376 ± 69	401 ± 63 *	0.026
eGFR, mL/s/1.73 m^2^	1.73 ± 0.22	1.73 ± 0.22	1.71 ± 0.22	1.75 ± 0.19	0.782
U-alb/crea, mg/mmoL	0.4 (0.2; 0.9)	0.4 (0.2; 1.0)	0.4 (0.2; 0.7)	0.3 (0.2; 0.7)	0.457
CRP, mg/L	0.7 (0.2; 2.5)	0.7 (0.2; 2.6)	0.6 (0.2; 2.2)	0.8 (0.3; 2.8)	0.374
Adiponectin, mg/L	12.2 (6.1; 24.3)	12.7 (6.4; 25.2)	11.7 (5.9; 23.2)	10.1 (5.0; 20.7)	0.049 ^a^
sRAGE, ng/L	1549 (1106; 2170)	1574 (1113; 2225)	1512 (1100; 2080) *	1480 (1081; 2027)	0.299
esRAGE, ng/L	302 (195; 469)	305 (196; 476)	306 (201; 465) **	280 (181; 433)	0.354
cRAGE, ng/L	1234 (876; 1736)	1255 (883; 1784) *	1193 (861; 1652) *	1191 (873; 1623)	0.188
TBARs (μmol/L)	1.32 (0.76; 2.31)	1.30 (0.74; 2.27)	1.35 (0.78; 2.33)	1.43 (0.81; 2.53)	0.348
Homocysteine, μmol/L	11.4 (7.7; 16.9)	11.3 (7.6; 16.9)	11.2 (8.0; 15.8)	12.1 (7.9; 18.7)	0.393
Leukocytes, 10^9^/L	6.6 ± 1.6	6.6 ± 1.5	6.6 ± 1.5	6.8 ± 1.4	0.613
**Prevalence**					***p_Chi_***
**Elevated**					
Systolic BP, *n* (%)	167 (31.8)	-	108 (94.7)	59 (96.7)	0.827
Diastolic BP, *n* (%)	49 (9.3)	-	24 (21.1)	25 (41.0)	0.003
Waist/height, *n* (%)	162 (30.9)	87 (24.9)	41 (36.0)	34 (55.7)	<0.001
Glucose, *n* (%)	80 (15.2)	53 (15.1)	18 (15.8)	9 (14.8)	0.980
Insulin, *n* (%)	96 (18.3)	62 (17.7)	21 (18.4)	13 (21.3)	0.798
Triacylglycerols, *n* (%)	68 (13.0)	46 (13.1)	14 (12.3)	8 (13.15)	0.971
Atherogenic index, *n* (%)	108 (20.6)	73 (20.9)	22 (19.3)	13 (21.3)	0.927
Uric acid, *n* (%)	164 (31.2)	105 (30.0)	35 (30.7)	24 (39.3)	0.345
CRP, *n* (%)	86 (16.4)	61 (17.4)	16 (14.0)	9 (14.8)	0.652
Low HDL-C, *n* (%)	176 (33.5)	127 (35.4)	31 (27.2)	18 (29.5)	0.158
Metabolic syndrome, *n* (%)	50 (9.5)	12 (3.4)	20 (17.5)	18 (29.5)	<0.001

BP—blood pressure. ANOVA—analysis of variance. BMI—body mass index. QUICKI—quantitative insulin sensitivity check index. HDL-C—high- density lipoprotein cholesterol. LDL-C—low-density lipoprotein cholesterol. Met—metabolic. eGFR—estimated glomerular filtration rate. U-alb/crea—urinary albumin-to-creatinine ratio. CRP –C-reactive protein. sRAGE—soluble receptor for advanced glycation end products. esRAGE—endogenous secretory RAGE. cRAGE—cleaved RAGE. TBARs—thiobarbituric acid reactive substances. Chi: chi-square. Data are presented as mean ± SD, or data not fitting to normal distribution that were logarithmically transformed prior to statistical analysis and are given as geometric mean (−1SD; +1SD range) of the back-transformed log data, or as counts (percentage). ^a^: The post-hoc tests did not localize the between-group difference. *, **, ***: *p* < 0.05, 0.01, 0.001, respectively, vs. normotensive subjects. ^+^, ^++^, ^+++^: *p* < 0.05, 0.01, 0.001, vs. subjects with high normal blood pressure.

**Table 5:** Characteristics of males not presenting cardiometabolic abnormalities.

**Variable**	**All**	**P_t*-test*_ vs. MA+**	**Normotensive**	**High Normal BP**	**Hypertensive**	***p_ANOVA_***
*n*	697		541	106	50	
Age, years	17.4 ± 1.2	0.036	17.3 ± 1.1	17.5 ± 1.2	17.6 ± 1.0	0.140
Systolic BP, mmHg	121 ± 12	<0.001	117 ± 8	133 ± 4 ***	147 ± 9 ***^,+++^	<0.001
Diastolic BP, mmHg	72 ± 8	<0.001	70 ± 6	76 ± 6 ***	82 ± 8 ***^,+++^	<0.001
Heart rate, beats/min	77 ± 13	0.018	76 ± 12	76 ± 14	84 ± 16 ***^,++^	0.001
Height, cm	179.1 ± 6.7	0.977	178.5 ± 6.7	180.4 ± 6.2 *	182.4 ± 6.1 ***	<0.001
Weight, kg	69.7 ± 9.7	<0.001	68.1 ± 9.3 ^ooo^	73.8 ± 8.6 ***^,ooo^	78.2 ± 9.7 ***^,+,ooo^	<0.001
Waist, cm	76.1 ± 5.5	<0.001	75.4 ± 5.4 ^ooo^	77.8 ± 5.1 ***^,ooo^	79.6 ± 5.8 ***^,ooo^	<0.001
BMI, kg/m^2^	21.7 ± 2.5	<0.001	21.4 ± 2.4 ^ooo^	22.7 ± 2.3 ***^,ooo^	23.5 ± 2.5 ***^,ooo^	<0.001
Waist/height	0.43 ± 0.03	<0.001	0.42 ± 0.03 ^ooo^	0.43 ± 0.03 *^,ooo^	0.44 ± 0.03 **^,ooo^	0.001
TBF, %	15.0 ± 5.1	<0.001	14.5 ± 5.1 ^ooo^	16.4 ± 5.0 **^,ooo^	17.4 ± 4.6 ***^,ooo^	<0.001
FFM, kg	56.7 ± 5.8	<0.001	55.7 ± 5.6 ^ooo^	59.0 ± 5.0 ***^,ooo^	61.5 ± 5.5 ***^,+,ooo^	<0.001
Glucose, mmol/L	4.9 ± 0.3	<0.001	4.8 ± 0.3 ^ooo^	4.9 ± 0.3	4.9 ± 0.3	0.082
Insulin, μIU/mL	8.1 (5.5; 11.8)	<0.001	7.9 (5.4; 11.4) ^ooo^	8.6 (5.9; 12.5) ^ooo^	9.5 (6.5; 13.9) **^,oo^	0.001
QUICKI	0.352 ± 0.022	<0.001	0.353 ± 0.022 ^ooo^	0.348 ± 0.021 ^ooo^	0.343 ± 0.020 **^,o^	0.001
Cholesterol, mmol/L	3.77 ± 0.62	0.002	3.75 ± 0.61	3.89 ± 0.62	3.84 ± 0.67	0.070
HDL-C, mmol/L	1.32 ± 0.20	0.001	1.32 ± 0.20 ^ooo^	1.32 ± 0.19 ^ooo^	1.34 ± 0.20 ^ooo^	0.786
LDL-C, mmol/L	2.12 ± 0.53	<0.001	2.09 ± 0.53	2.22 ± 0.53	2.15 ± 0.59	0.078
Non-HDL-C, mmol/L	2.46 ± 0.57	<0.001	2.43 ± 0.56 ^ooo^	2.57 ± 0.59 *^,oo^	2.50 ± 0.64 ^o^	0.052
TAG, mmol/L	0.71 (0.51; 0.99)	<0.001	0.70 (0.50; 0.97) ^ooo^	0.73 (0.51; 1.05) ^oo^	0.74 (0.52; 1.04) ^oo^	0.339
Atherogenic index	−0.26 ± 0.16	<0.001	−0.27 ± 0.16 ^ooo^	−0.25 ± 0.17 ^ooo^	−0.25 ± 0.17^ooo^	0.521
Continuous Metabolic score	3.29 ± 0.60	<0.001	3.22 ± 0.56 ^oo^	3.48 ± 0.63 ***^,ooo^	3.67 ± 0.67 ***^,ooo^	<0.001
Met score -BP	2.36 ± 0.58	<0.001	2.32 ± 0.55 ^oo^	2.46 ± 0.63 ^ooo^	2.54 ± 0.66 *^,ooo^	0.003
Uric acid, μmol/L	336 ± 45	<0.001	334 ± 46 ^ooo^	340 ± 40 ^ooo^	348 ± 37 ^ooo^	0.045 ^a^
eGFR, mL/s/1.73 m^2^	1.75 ± 0.23	0.645	1.75 ± 0.23	1.75 ± 0.23	1.70 ± 0.21	0.177
U-alb/crea, mg/mmol	0.4 (0.2; 1.0)	0.149	0.4 (0.2; 1.0)	0.4 (0.1; 0.9)	0.3 (0.1; 0.9)	0.287
CRP, mg/L	0.7 (0.2; 2.5)	<0.001	0.7 (0.2; 2.6) ^ooo^	0.6 (0.2; 2.2) ^ooo^	0.8 (0.3; 2.8) ^oo^	0.733
Adiponectin, mg/L	12.2 (6.1; 24.3)	0.031	11.7 (6.4; 25.2)	11.7 (5.9; 23.2)	10.1 (5.0; 20.7)	0.355
sRAGE, ng/L	1549 (1106; 2170)	0.044	1574 (1113; 2225)	1512 (1100; 2080)	1480 (1081; 2027) *	0.023
esRAGE, ng/L	302 (195; 469)	0.019	305 (196; 476)	306 (201; 465)	280 (181; 433) *	0.014
cRAGE, ng/L	1234 (876; 1736)	0.093	1255 (883; 1784)	1193 (861; 1652)	1191 (873; 1623) *	0.028
TBARs (μmol/L)	1.32 (0.76; 2.31)	0.994	1.30 (0.74; 2.27)	1.35 (0.78; 2.33)	1.43 (0.81; 2.53)	0.145
Hcy, μmol/L	11.4 (7.7; 16.9)	0.479	11.3 (7.6; 16.9)	11.2 (8.0; 15.8)	12.1 (7.9; 18.7)	0.882
Leukocytes, 10^9^/L	6.2 ± 1.3	<0.001	6.1 ± 1.3 ^ooo^	6.1 ± 1.1 ^o^	6.5 ± 1.5	0.091
**Prevalence**						***p_Chi_***
eSBP	147 (21.1)	<0.001	-	100 (94.3)	47 (94.0)	0.777
eDBP	34 (4.9)	0.002	-	24 (22.6)	10 (20.0)	0.709

BP—blood pressure. SBP: systolic blood pressure. DBP: diastolic blood pressure. MA+: metabolic abnormalities presenting males. ANOVA—analysis of variance. BMI—body mass index. TBF: total body fat. FFM: fat-free mass. QUICKI—quantitative insulin sensitivity check index. HDL-C—high-density lipoprotein cholesterol. LDL-C—low-density lipoprotein cholesterol. TAG: triacylglycerols. Met—metabolic. Met-BP: continuous metabolic score without systolic BP component. eGFR—estimated glomerular filtration rate. U-alb/crea—urinary albumin-to-creatinine ratio. CRP—C-reactive protein. sRAGE—soluble receptor for advanced glycation end products. esRAGE—endogenous secretory RAGE. cRAGE—cleaved RAGE. TBARs—thiobarbituric acid reactive substances. Hcy—homocysteine. e—elevated. Chi: chi-square. Data are presented as mean ± SD. Data not fitting to normal distribution were logarithmically transformed prior to statistical analysis and are given as a geometric mean (−1SD; +1SD range) of the back-transformed log data, or as counts (percentage). *, **, ***: *p* < 0.05, 0.01, 0.001, respectively, vs. normotensive subjects. ^+^, ^++^, ^+++^: *p* < 0.05, 0.01, 0.001, vs. subjects with high normal blood pressure. ^o, oo, ooo^: *p* < 0.05, 0.01, 0.001, respectively, vs. males in the corresponding blood pressure category presenting cardiometabolic abnormalities.

**Table 6:** Characteristics of females.

**Variable**	**Normotensive**	**High Normal BP**	**Hypertensive**	***p*** ***_K-W_***
*n*	1 255	53	13	
Age, years	17.5 ± 1.2	18.0 ± 1.4	17.9 ± 1.0	0.018
Systolic BP, mmHg	106 ± 8	123 ± 7	130 ± 6	<0.001
Diastolic BP, mmHg	70 ± 7	85 ± 5	94 ± 3	<0.001
Heart rate, beats/min	81 ± 12	89 ± 16	89 ± 20	<0.001
Height, cm	165.6 ± 6.2	165.8 ± 7.0	168.2 ± 7.8	0.424
Weight, kg	59.9 ± 10.3	63.4 ± 11.2	69.0 ± 21.0	0.014
Waist, cm	71.4 ± 7.7	74.5 ± 9.2	79.5 ± 15.5	0.006
BMI, kg/m^2^	21.8 ± 3.4	23.0 ± 3.6	24.5 ± 7.7	0.032
Waist/height	0.43 ± 0.05	0.45 ± 0.06	0.47 ± 0.10	0.037
Total body fat, %	30.2 ± 6.8	32.3 ± 7.2	33.0 ± 11.4	0.040
Fat-free mass, kg	39.2 ± 4.6	40.6 ± 5.2	42.5 ± 7.2	0.013
Glucose, mmol/L	4.7 ± 0.4	4.7 ± 0.4	4.6 ± 0.3	0.960
Insulin, μIU/mL	11.1 ± 6.3	13.1 ± 9.3	13.4 ± 8.6	0.149
QUICKI	0.344 ± 0.025	0.338 ± 0.028	0.343 ± 0.045	0.178
Cholesterol, mmol/L	4.25 ± 0.76	4.41 ± 0.66	4.36 ± 0.61	0.019
HDL-C, mmol/L	1.52 ± 0.30	1.50 ± 0.34	1.64 ± 0.32	0.257
LDL-C, mmol/L	2.33 ± 0.61	2.47 ± 0.50	2.56 ± 0.50	0.043
Non-HDL-C, mmol/L	2.73 ± 0.69	2.91 ± 0.62	2.89 ± 0.68	0.018
Triacylglycerols, mmol/L	0.88 ± 0.39	0.97 ± 0.50	1.04 ± 0.47	0.344
Atherogenic index	−0.27 ± 0.20	−0.23 ± 0.23	−0.24 ± 0.25	0.550
Continuous metabolic score	3.52 ± 0.89	4.01 ± 1.26	4.06 ± 1.33	<0.001
Met score without SBP component	2.70 ± 0.87	3.06 ± 1.27	3.06 ± 1.33	<0.001
Uric acid, μmol/L	257 ± 51	260 ± 53	285 ± 43	0.109
eGFR, mL /s/1.73 m^2^	1.80 ± 0.23	1.80 ± 0.23	1.85 ± 0.26	0.891
U-alb/crea, mg/mmol	1.1 ± 4.2	0.7 ± 1.0	5.2 ± 14.6	0.417
CRP, mg/L	1.1 ± 1.5	1.7 ± 2.2	1.3 ± 1.8	0.231
Adiponectin, mg/L	23.7 ± 19.0	17.5 ± 10.1	18.3 ± 9.5	0.222
sRAGE, ng/L	1639 ± 596	1585 ± 496	1489 ± 455	0.665
esRAGE, ng/L	340 ± 155	332 ± 138	318 ± 120	0.904
cRAGE, ng/L	1299 ± 494	1253 ± 401	1171 ± 372	0.519
TBARs (μmol/L)	1.53 ± 0.82	1.56 ± 0.78	1.52 ± 0.90	0.921
Homocysteine, μmol/L	9.9 ± 3.4	10.1 ± 3.0	9.9 ± 3.1	0.755
Leukocytes, 10^9^/L	6.8 ± 1.8	6.9 ± 1.8	7.4 ± 1.6	0.388
**Prevalence**				***p*** **_Chi_**
**Elevated**				
Systolic BP, *n* (%)	-	13 (24.5)	7 (53.8)	0.085
Diastolic BP*, n* (%)	-	44 (83.0)	13 (100)	0.251
Waist/height, *n* (%)	113 (9.0)	8 (15.1)	5 (38.5)	0.006
Glucose, *n* (%)	25 (2.0)	2 (3.8)	0	0.829
Insulin, *n* (%)	74 (5.9)	4 (7.5)	3 (23.1)	0.137
Triacylglycerols, *n* (%)	56 (5.1)	7 (13.2)	2 (15.4)	0.020
Atherogenic index, *n* (%)	45 (3.9)	5 (9.4)	0	0.191
Uric acid, *n* (%)	62 (4.9)	6 (11.3)	2 (15.4)	0.136
CRP, *n* (%)	130 (10.4)	7 (13.2)	2 (15.4)	0.681
Low HDL-C, *n* (%)	285 (22.7)	16 (30.2)	3 (23.1)	0.448
Metabolic syndrome, *n* (%)	5 (0.4)	11 (20.8)	3 (23.1)	<0.001

BP—blood pressure. K-W: Kruskal-Wallis test. BMI—body mass index. QUICKI—quantitative insulin sensitivity check index. HDL-C—high- density lipoprotein cholesterol. LDL-C—low-density lipoprotein cholesterol. MetSy—metabolic syndrome. eGFR—estimated glomerular filtration rate. U-alb/crea—urinary albumin-to-creatinine ratio. CRP—C-reactive protein. sRAGE—soluble receptor for advanced glycation end products. esRAGE—endogenous secretory RAGE. cRAGE—cleaved RAGE. TBARs—thiobarbituric acid reactive substances. Chi: chi-square. Data are presented as mean ± SD or as counts (percentage).

## Supplementary Materials

The following are available online at https://www.mdpi.com/1660-4601/17/10/3612/s1. Table S1: Characteristics of females presenting cardiometabolic abnormalities. Table S2: Characteristics of females not presenting cardiometabolic abnormalities.
