# Supplementary material for: Sex Differences in Association of Elevated Blood Pressure with Variables Characterizing Cardiometabolic Risk in Young Subjects with or Without Metabolic Abnormalities
Source: Int J Environ Res Public Health. 2020 May 21;17(10):3612. doi: 10.3390/ijerph17103612 (PMC7277167; doi:10.3390/ijerph17103612)
Supplement: Supplementary file 1 [file ijerph-17-03612-s001.pdf]

Supplemental material to: Sex differences in association of elevated blood pressure with variables characterizing cardiometabolic risk in young subjects with or without metabolic abnormalities, by

Šebeková K[1], Gurecká R[1,2], Csongová M[1], Koborová I[1], Šebek J[3]

[1]Institute of Molecular BioMedicine, Faculty of Medicine, Comenius, University, Bratislava, Slovakia; [2]Institute of Medical Physics, Biophysics, Informatics and Telemedicine, Faculty of Medicine, Comenius University, Bratislava, Slovakia; [3]Institute of Materials & Machine Mechanics, Slovak Academy of Sciences, Bratislava, Bratislava, Slovakia

Supplemental Table S1: Characteristics of females presenting cardiometabolic abnormalities

	All	Normotensive	High normal BP	Hypertensive
N	558	525 (94.1%)	25 (4.5%)	8 (1.4%)
Age, years	17.5±1.2	17.5±1.2	18.0±1.4	17.9±1.0
Systolic BP, mm Hg	108±9	107±8	123±7	129±7
Diastolic BP, mm Hg	71±8	70±7	85±4	94±32
Heart rate, beats/min	81±13	81±12	89±18	75±23
Height, cm	165.1±6.1	165.2±6.1	164.0±5.9	168.1±8.4
Weight, kg	63.1±12.4	62.8±12.2	67.9±11.3	77.7±2.8
Waist, cm	74.5±9.6	74.2±9.3	79.2±9.37	87.2±15.1
BMI, kg/m^2	23.1±4.2	23.0±4.1	25.2±4.0	27.7±8.3
Waist/height	0.45±0.06	0.45±0.06	0.48±0.06	0.52±0.10
Total body fat, %	32.6±7.4	32.4±7.3	35.8±7.2	38.0±11.6
Fat-free mass, kg	40.3±5.1	40.1±5.1	41.4±4.0	45.6±7.7
Glucose, mmol/l	4.7±0.4	4.7±0.4	4.7±0.5	4.6±0.4
Insulin, µIU/ml	*13.4±8.6*	*13.3±8.4*	*16.6±12.1*	*15.8±10.3*
QUICKI	0.336±0.028	0.337±0.027	0.327±0.026	0.342±0.057
Cholesterol, mmol/l	4.19±0.81	4.17±0.82	4.39±0.85	4.68±0.52
HDL-C, mmol/l	1.37±0.31	1.37±0.30	1.34±0.43	1.55±0.38
LDL-C, mmol/l	2.36±0.62	2.35±0.62	2.50±0.55	2.57±0.48
Non-HDL-C, mmol/l	2.82±0.72	2.80±0.72	3.05±0.70	3.12±0.50
Triacylglycerols, mmol/l	*1.01±0.48*	*0.99±0.48*	*1.19±0.63*	*1.23±0.43*
Atherogenic index	-0.17±0.20	-0.17±0.20	-0.10±0.24	-0.12±0.23
Continuous MetSy score	4.15±1.02	4.11±0.98	4.80±1.39	4.71±1.33
MetSy score without SBP component	3.32±1.00	3.39±0.97	3.85±1.40	3.72±1.31
Uric acid, mmol/l	269±58	267±58	288±57	302±43
eGFR, ml/s/1.73m^2	1.82±0.24	1.82±0.24	1.86±0.23	1.90±0.30
U-alb/crea, mg/mmol	1.2±1.0	*1.0±4.6*	*2.0±0.3*	*2.1±0.4*
hsCRP, mg/l	1.8±2.1	*1.7±2.0*	*2.7±3.8*	*2.0±2.1*
Adiponectin, mg/l	*19.1±15.6*	*19.3±15.8*	*15.6±10.3*	*17.4±11.5*
sRAGE, ng/l	*1574±597*	*1584±606*	*1384±411*	*1486±427*
esRAGE, ng/l	*318±142*	*319±143*	*296±134*	*332±136*
cRAGE, ng/l	*1255±491*	*1265±499*	*1088±317*	*1155±304*
TBARs, µmol/l	*1.49±0.81*	*1.49±0.82*	*1.32±0.56*	*1.55±0.84*
Hcy, µmol/l	*10.0±3.5*	*10.0±3.6*	*10.0±2.5*	*9.8±4.0*

Leukocytes, 10^9/l	7.2±1.9	7.2±1.9	7.5±2.0	7.7±1.9
**Prevalence**				
Elevated:				
- Systolic BP, n (%)	11 (1.9)	--	7 (28.0)	4 (50.0)
- Diastolic BP n (%)	29 (5.2)	--	21 (84.0)	8 (100)
- Waist/height, n (%)	126 (22.6)	113 (21.5)	8 (32.0)	5 (62.5)
- Glucose, n (%)	27 (4.8)	25 (4.8)	2 (8.0)	0
- Insulin, n (%)	81 (14.5)	74 (14.1)	4 (16.0)	3 (37.5)
- Triacylglycerols, n (%)	65 (11.6)	56 (10.7)	7 (28.0)	2 (25.0)
- Atherogenic index, n (%)	50 (9.0)	45 (8.6)	5 (20.0)	0
- Uric acid, n (%)	70 (12.5)	62 (11.8)	6 (24.0)	2 (25.0)
- hsCRP, n (%)	139 (24.5)	130 (24.8)	7 (28.0)	2 (25.0)
Low HDL-C, n (%)	304 (54.4)	285 (54.3)	16 (64.0)	3 (37.5)
Metabolic syndrome, n (%)	19 (3.4)	5 (1.0)	11 (44.0)	3 (37.5)

BP – blood pressure, BMI – body mass index, QUICKI – quantitative insulin sensitivity check index, HDL-C – high- density lipoprotein cholesterol, LDL-C – low-density lipoprotein cholesterol, MetSy – metabolic syndrome, SBP – systolic blood pressure, eGFR – estimated glomerular filtration rate, U-alb/crea - urinary albumin-to-creatinine ratio, hsCRP – high sensitive C-reactive protein, sRAGE – soluble receptor for advanced glycation end products, esRAGE – endogenous secretory RAGE, cRAGE – cleaved RAGE, TBARs - thiobarbituric acid reactive substances, Hcy – homocysteine, data are presented as mean±SD or as counts (percentage), data not fitting to normal distribution (given in Italics) were logarithmically transformed prior to statistical analysis

Suplemental Table S2: Characteristics of females not presenting cardiometabolic abnormalities

	**All**	**$P_{t\text{-}test}$ vs. MA+**	**Normotensive**	**High normal BP**	**Hypertensive**
N	763		730 (95.7%)	28 (3.7%)	5 (0.6%)
Age, years	17.5±1.2	0.912	17.5±1.2	17.9±1.5	18.0±1.0
Systolic BP, mm Hg	107±9	**0.013**	106±8	124±7	132±4
Diastolic BP, mm Hg	70±7	**0.004**	69±7	84±5	93±2
Heart rate, beats/min	81±13	0.277	80±12	89±19	89±20
Height, cm	166.0±6.3	**0.019**	165.9±6.3	167.6±7.7	168.4±7.7
Weight, kg	57.9±8.1	**<0.001**	57.8±8.0	60.5±11.5	55.0±4.0
Waist, cm	69.5±5.5	**<0.001**	69.5±5.4	71.0±7.8	67.1±3.1
BMI, kg/m^2	21.0±2.4	**<0.001**	21.0±2.4	21.4±2.6	19.5±1.6
Waist/height	0.42±0.03	**<0.001**	0.42±0.03	0.42±0.04	0.40±0.01
Total body fat, %	28.6±5.9	**<0.001**	28.6±5.9	29.6±6.2	25.0±5.0
Fat-free mass, kg	38.5±4.1	**<0.001**	38.4±4.0	39.9±6.0	37.7±2.6
Glucose, mmol/l	4.6±0.3	**<0.001**	4.6±0.3	4.7±0.3	4.7±0.2
Insulin, µIU/ml	9.5±3.5	***<0.001***	*9.5±3.5*	*10.4±4.3*	*9.7±2.8*
QUICKI	0.350±0.022	**<0.001**	0.350±0.022	0.346±0.025	0.345±0.014
Cholesterol, mmol/l	4.31±0.71	**0.005**	4.30±0.71	4.39±0.58	4.66±0.80
HDL-C, mmol/l	1.63±0.25	**<0.001**	1.63±0.25	1.60±0.22	1.77±0.18

LDL-C, mmol/l	2.32±0.61	0.230	2.31±0.61	2.43±0.49	2.55±0.60
Non-HDL-C, mmol/l	2.68±0.65	**<0.001**	2.67±0.66	2.78±0.56	2.89±0.68
TAG, mmol/l	*0.81±0.30*	***<0.001***	*0.79±0.29*	*0.78±0.25*	*0.75±0.41*
Atherogenic index	-0.34±0.17	**<0.001**	-0.34±0.17	-0.33±0.15	-0.42±0.18
Continuous MetSy score	3.10±0.47	**<0.001**	3.09±0.47	3.31±0.51	3.03±0.25
MSS-TK	2.28±0.46	**<0.001**	2.28±0.46	2.36±0.49	2.02±0.25
Uric acid, mmol/l	249±43	**<0.001**	249±43	241±40	258±31
eGFR, ml/s/1.73m^2	1.79±0.22	0.091	1.80±0.22	1.75±0.21	1.76±0.16
U-alb/crea, mg/mmol	*1.2±4.3*	*0.624*	*1.1±4.0*	*0.9±1.4*	*1.9±0.2*
hsCRP, mg/l	*0.6±0.7*	***<0.001***	*0.6±0.7*	*0.7±0.7*	*0.2±0.2*
Adiponectin, mg/l	*26.6±23.2*	***<0.001***	*26.9±23.6*	*19.1±8.9*	*19.5±6.9*
sRAGE, ng/l	*1681±583*	***<0.001***	*1679±587*	*1764±503*	*1494±548*
esRAGE, ng/l	*356±161*	***<0.001***	*356±162*	*364±135*	*296±101*
cRAGE, ng/l	*1326±486*	***0.001***	*1324±489*	*1400±417*	*1198±502*
TBARs, µmol/l	*1.56±0.82*	*0.092*	*1.55±0.82*	*1.68±0.85*	*1.49±1.14*
Hcy, µmol/l	*9.9±3.2*	*0.907*	*9.9±3.2*	*10.2±3.4*	*9.9±0.5*
Leukocytes, 10^9/l	6.6±1.6	**<0.001**	6.6±1.7	6.4±1.2	7.1±1.1
**Prevalence**					
eSBP	9 (1.2)	0.244	--	6 (21.4)	3 (60.0)
eDBP	28 (3.7)	0.177	--	23 (82.1)	5 (100.0)

BP – blood pressure, BMI – body mass index, QUICKI – quantitative insulin sensitivity check index, HDL-C – high- density lipoprotein cholesterol, LDL-C – low-density lipoprotein cholesterol, MetSy – metabolic syndrome, SBP – systolic blood pressure, eGFR – estimated glomerular filtration rate, U-alb/crea - urinary albumin-to-creatinine ratio, hsCRP – high sensitive C-reactive protein, sRAGE – soluble receptor for advanced glycation end products, esRAGE – endogenous secretory RAGE, cRAGE – cleaved RAGE, TBARs - thiobarbituric acid reactive substances, Hcy – homocysteine, eSBP - elevated systolic BP, eDBP – elevated diastolic BP, data are presented as mean±SD or as counts (percentage), data not fitting to normal distribution (given in Italics) were logarithmically transformed prior to statistical analysis
